# Is the stereoisomer R-MDMA a safer version of MDMA?

**DOI:** 10.1038/s41386-024-02009-8

**Published:** 2024-10-24

**Authors:** Gillinder Bedi

**Affiliations:** Centre for Youth Mental Health, University of Melbourne, and Orygen, Melbourne, Australia

**Keywords:** Human behaviour, Translational research

There are many outstanding questions about the potential therapeutic use of 3,4-methylenedioxymethamphetamine (MDMA) as an adjunct to psychotherapy for treatment of psychiatric illness—particularly given the Food and Drug Administration’s recent decision to decline approval of MDMA-assisted psychotherapy for Post-Traumatic Stress Disorder. One key question that has received little attention in the clinical literature is whether small variations in the structural or chemical makeup of MDMA can yield changes to the effect profile that may positively shift the risk: benefit profile of this compound.

In a within-subjects controlled laboratory study, Straumann et al. [1] present the first human data collected using modern experimental methods to investigate this question. Previously published human laboratory studies and clinical trials of MDMA have employed a racemic mixture containing equal parts of the two enantiomers of MDMA, S(+)- and R(-)-MDMA. Yet, preclinical work indicates that there may be clinically relevant differences in the pharmacodynamic and pharmacokinetic properties of these enantiomers. Of note, in rodents the S-enantiomer produces more psychostimulant-like effects as demonstrated in a discriminative stimulus paradigm [2], and more potently increases dopaminergic neurotransmission [3], than R-MDMA. Conversely, R-MDMA was shown in a discriminative stimulus experiment to have more psychedelic-like effects in rodents than S-MDMA [2], with lower potential for neurotoxic or adverse thermoregulatory effects [4]. Together with the higher abuse liability of psychostimulants relative to psychedelics, these findings suggest that R-MDMA could be a safer version of MDMA for clinical use.

Straumann et al. [1] investigate this possibility using a double-blind, placebo controlled, cross-over design to directly compare the effects of R-MDMA (125, 250 mg), S-MDMA (125 mg), racemic MDMA (125 mg), and placebo across a range of outcome domains, with subjective stimulation and psychedelic effects the primary outcomes. They hypothesise that S-MDMA will produce greater subjective stimulation than R-MDMA, and that R-MDMA will produce greater psychedelic-like effects relative to S-MDMA.

The racemic MDMA dose was selected to approximate doses commonly used in clinical trials. Doses of S- and R-MDMA were based on preclinical evidence indicating similar potency for racemic and S-MDMA, with lower potency in R-MDMA. Participants were 24 healthy adults, with equal representation of women and men. Almost all participants (n = 22) had prior exposure to cannabis, most (*n* = 18) had used other illicit drugs, and 11 had used MDMA (there was no effect of prior MDMA use on the study endpoints). With the exception of cannabis, lifetime use of any illicit drug on more than 20 occasions was exclusionary.

Although—as hypothesised—S-MDMA increased subjective ratings of feeling stimulated relative to both doses of R-MDMA, it also increased several other subjective mood states compared to R-MDMA, and some relative to racemic MDMA. Of note given the putative mechanistic role of MDMA’s positive social effects in MDMA-assisted psychotherapy [5], S-MDMA increased ratings of several prosocial mood states compared to the R isomer.

Also contrary to expectations, R-MDMA did not produce stronger psychedelic-like effects than S-MDMA or the racemic compound, with equivalent effects in this domain across R-MDMA (250 mg), S-MDMA and racemic MDMA (and lower such effects after R-MDMA 125 mg). All active conditions caused similar transient adverse effects during the 10-h sessions and in the period immediately afterwards; 1–3 days after S-MDMA, self-rated depressive symptoms and overall psychological distress were higher than after placebo, with no other differences across conditions. Neuroendocrine responses were also preferentially increased after S-MDMA, with higher plasma oxytocin and cortisol levels in the S-MDMA condition compared to both R-MDMA (250 mg) and racemic MDMA, and higher plasma prolactin relative to the racemic compound. The study revealed substantial differences in the pharmacokinetic parameters of the stereoisomers, with the elimination half-life of R-MDMA (14 h for 250 mg) some three times longer than that of S-MDMA (4.1 h).

What is to be gleaned from these results? Somewhat disappointingly, overall results from this first study suggest that the doses employed might not be equivalent, with the S-MDMA dose likely marginally too high, and the highest R-MDMA dose likely too low. It therefore remains unclear whether the differences observed are due to qualitative differences between the isomers or to dose-related effects. The authors estimate that closer dose equivalence would be obtained with doses around 125 mg for racemic MDMA, 100 mg for S-MDMA and 300 mg for R-MDMA. Hopefully, a comparable study using these doses will be forthcoming from this group in the future.

As the first study to directly compare the isomers of MDMA with the racemic compound in humans, this study had several strengths—including the highly-controlled within-subjects design and comprehensive measurement of a range of outcome measures over the acute session and in the hours and days afterwards. There were also some limitations beyond the apparent dose inequivalence. In particular, it is unfortunate that despite the sample comprising equal numbers of males and females—providing a rare opportunity to investigate sex differences in acute responding to MDMA and its stereoisomers—the lack of weight-based dosing means that the differences in effects between men and women observed are likely attributable to differences in weight. Although weight-based dosing adds to the practical complexity of conducting studies of this type, it is to be hoped that future studies will better assess sex differences in MDMA’s effects by investing resources in weight-based dosing.

Overall results of this study do not support the hypothesis that R-MDMA could be safer than racemic or S-MDMA for clinical use – with reduced psychostimulant effects but retaining prosocial ‘empathogenic’ properties implicated in the therapeutic effects of MDMA. Yet, it is to be hoped that this initial study will spur further research investigating whether small variations in MDMA—such as use of single enantiomers rather than racemic MDMA—could reduce safety concerns while retaining or enhancing effects implicated in the therapeutic potential of this compound. Combined with robust investigation of optimal approaches to the psychotherapy component of MDMA-assisted psychotherapy, such a research programme could point the way towards the next generation of approaches to the psychotherapeutic use of MDMA.
